# Morphological Abnormalities and Gene Expression Changes Caused by High Incubation Temperatures in Zebrafish Xenografts with Human Cancer Cells

**DOI:** 10.3390/genes12010113

**Published:** 2021-01-19

**Authors:** Pablo Cabezas-Sainz, Carlos Coppel, Alba Pensado-López, Pedro Fernandez, Laura Muinelo-Romay, Rafael López-López, Juan A. Rubiolo, Laura Sánchez

**Affiliations:** 1 Department of Zoology, Genetics and Physical Anthropology, Universidade de Santiago de Compostela, Campus de Lugo, 27002 Lugo, Spain; pablo.cabezas@usc.es (P.C.-S.); carloscoppel13@gmail.com (C.C.); alba.pensado.lopez@rai.usc.es (A.P.-L.); pedro.fernandez.garcia@rai.usc.es (P.F.); 2 Genomic Medicine Group, Center for Research in Molecular Medicine and Chronic Diseases (CiMUS), Universidade de Santiago de Compostela, 15782 Santiago de Compostela, Spain; 3 Liquid Biopsy Analysis Unit, Oncomet, Health Research Institute of Santiago de Compostela (IDIS), 15706 Santiago de Compostela, Spain; lmuirom@gmail.com; 4 Centro de Investigación Biomédica en Red de Cáncer (CIBERONC), 29029 Madrid, Spain; 5 Translational Laboratory, Medical Oncology Department, Complexo Hospitalario Universitario de Santiago de Compostela/SERGAS, 15706 Santiago de Compostela, Spain; rafael.lopez.lopez@sergas.es; 6 Preclinical Animal Models Group, Health Research Institute of Santiago de Compostela (IDIS), 15706 Santiago de Compostela, Spain

**Keywords:** zebrafish, development, xenotransplantation, incubation-temperature, gene-expression

## Abstract

Published studies show that most of the human cancer xenograft studies in zebrafish embryos have used incubation temperatures in the range of 32–34 °C for 3–6 days post-injection, trying to find a compromise temperature between the zebrafish embryos (28 °C) and the human injected cells (37 °C). While this experimental setup is widely used, a question remains: is possible to overcome the drawbacks caused by a suboptimal temperature for the injected cells? To clarify the effect of temperature and injected cells on the host, in this study, we analyzed the development and health of the last in response to different temperatures in the presence or absence of injected human cancer cells. Comparing different incubation temperatures (28, 34 and 36 °C), we determined morphological abnormalities and developmental effects in injected and non-injected embryos at different time points. Besides this, the expression of selected genes was determined by qPCR to determine temperature affected metabolic processes in the embryos. The results indicate that an incubation temperature of 36 °C during a period of 48 h is suitable for xenotransplantation without morphological or metabolic changes that could be affecting the host or the injected cells, allowing them to proliferate near their optimal temperature.

## 1. Introduction

Zebrafish embryos have been widely used as a model for cancer research since 2006 [1] by means of the xenograft technique. The procedure involves the injection of human cancer cells in different parts of zebrafish embryos, being the yolk and circulation injections at 48 h post-fertilization (hpf) the most widely used [2,3]. Additionally, xenograft studies can also be carried out in other sites of the embryos by cancer cell injection in the common cardinal vein, the brain, the perivitelline space, or the pericardial cavity at 48 hpf, 4 days post fertilization (dpf), or even in the adult fish [4,5]. The principal aim of this type of study is the determination of the injected cells’ fate and the effect of selected known/new compounds or chemotherapeutic drugs onto them [6]. Although many aspects of the technique had been studied in the last decade (site of injection, number of injected cells, image analysis, etc.) to develop and describe a model for the study of human cancer and its therapy [7], the incubation temperature of the embryos have not received much attention. Most of the studies have focused on the proliferation/invasion of cancer cells without the assessment of the incubation temperature effect on the host and the cell–host interaction underlying this process. While zebrafish embryos have an optimal developmental temperature of 28 °C [8], in xenograft experiments, a compromise temperature between host optimal growth temperature and that ideal for the human injected cells (37 °C) is used [9]. This temperature should be high enough for xenografted cells to grow properly, while allowing the embryos to stay alive and without malformations that compromise their survival during the experiment. The selected temperature for a given experiment will also differentially affect host gene expression. Previous works showed that a high incubation temperature-affected genes involved in stress response, immune response and development [10].

In xenograft, experiments temperature is important for the proliferation and migration of the injected cells. Considering this and the restrictions imposed by the host tolerance to temperature, a range of incubation temperatures (31 °C to 36 °C) have been assayed in xenografted zebrafish embryos to study injected cancer cells behavior, metabolic phenotype, or invasion capacity [11,12,13,14]. Results from in vitro studies on tumor cell proliferation, migration, and invasion at different temperatures (31–37 °C) suggest that temperatures closer to 37 °C are necessary to obtain reliable results when studying this type of cellular process [15].

The problem with xenograft studies in zebrafish at temperatures close to 37 °C is that, as described in other fish species [16], heat-induced teratogenic effects are expected. Teratogenic effects could be quite different at the temperature range of use in xenograft studies (31–37 °C) and should be determined to better understand the interactions, if any, between the host and the tumor cells. For this purpose, based on previous reports on temperature effects during zebrafish embryo development [17], we analyzed the teratogenic effects quantifying specific embryo malformations at different time points. While 34 °C or lower is the selected temperature in most of the zebrafish xenograft studies informed so far in the literature, we showed that these are feasible at 36 °C, reducing incubation time [7]. At 36 °C, we observed a higher injected cell proliferation rate when compared to lower temperatures, without significant host mortality. In this work, we focused on the quantification of malformations due to the differential temperature-induced teratogenic effects and determined their relation with embryo mortality to better characterize the zebrafish xenograft model at 36 °C.

Apart from embryo malformations, in xenograft experiments, temperature affects the expression of genes involved in several metabolic pathways involved in immune/stress response, inflammation, and development interfering with the overall state of the host [10,18]. While lacking an adaptive immune system, zebrafish embryos have an innate immune system at the time of tumor cell injection (48 hpf) [19,20]. Because of this, a differential immune and stress response at different temperatures could be interfering with the embryo reaction to the human injected cells. We analyzed this assaying the effect of temperature on selected genes.

## 2. Materials and Methods

### 2.1. Zebrafish Handling

One-year-old adult zebrafish (*Danio rerio*, wild-type, strain AB) were maintained at 28 °C in 30 L aquaria at a rate of 1 fish per liter of water, with a light–dark cycle of 14:10 hours. The aquarium is located in the veterinary facility of the University of Santiago de Compostela (Lugo, REGA code ES270280346401). Zebrafish embryos were obtained by mating adults according to previously described procedures [8]. Zebrafish care, use and treatment were performed in agreement with the European Parliament and Council Directive 2010/63/EU on the protection of animals used for scientific purposes and the Spain Royal Decree 53/2013 on animal welfare standards. Experimental protocols were approved by the Ethical Committee of the University of Santiago de Compostela (15,010/2015/001). After the experimental procedures, zebrafish embryos were euthanized with a tricaine (Sigma) overdose.

### 2.2. Cell Culture and GFP Labeling

The MCF7 human breast cancer cell line was obtained from the American Type Culture Collection (ATCC) and cultured using RPMI medium (GIBCO, Invitrogen—Carlsbad, CA, USA) containing 10% FBS (GIBCO, Invitrogen) and 1% Pen/Strep (GIBCO, Invitrogen), at 37 °C with 5% CO_2_ in a humidified atmosphere. For constitutive GFP labeling, cells were transduced using a lentiviral-driven GFP construct (Mission TurboGFP, SHC003V, Sigma—San Luis, Misouri, USA) following the manufacturer’s instructions. GFP-positive cells were selected 72 h post-infection, adding 10 µg/mL puromycin to the culture media.

### 2.3. Cancer Cell Injection in Zebrafish Embryos

Embryos were collected and grown at 28 °C in Petri dishes at a ratio of 50 embryos/plate. Two days post fertilization (dpf), embryos were dechorionized (if needed) and anesthetized with 0.003% tricaine (Sigma). GFP-labeled MCF7 breast cancer cells (10,000–20,000 cells/µL) were loaded into borosilicate glass capillary needles (1 mm O.D. × 0.75 mm I.D.; World Precision Instruments—Hitchin, Hertfordshire, UK) and injected at an average of 150–250 cells/embryo into the yolk sac using IM-31 electric microinjector (Narishige). We discarded embryos showing cells outside the yolk.

### 2.4. Incubation and Assays Conditions

Embryo incubation was performed at 28 °C, 34 °C and/or 36 °C in 140 mm × 20 mm Petri dishes (DeltaLab) at the ratio of 50 embryos/plate, using a minimum of two plates per replica, and preventing the plates from touching the metal parts of the incubator to avoid water overheating. Every 24 h, the egg water (salt dechlorinate tap water, SDTW) was renewed to account for evaporation, oxygen reduction or accumulation of excretion substances from the embryos. Embryos incubated for more than 6dpf were feed twice per day with Gemma 75 (Skretting). Each assay described below was performed in triplicate.

#### 2.4.1. Assay Starting at 0 hpf

**Figure 1:**
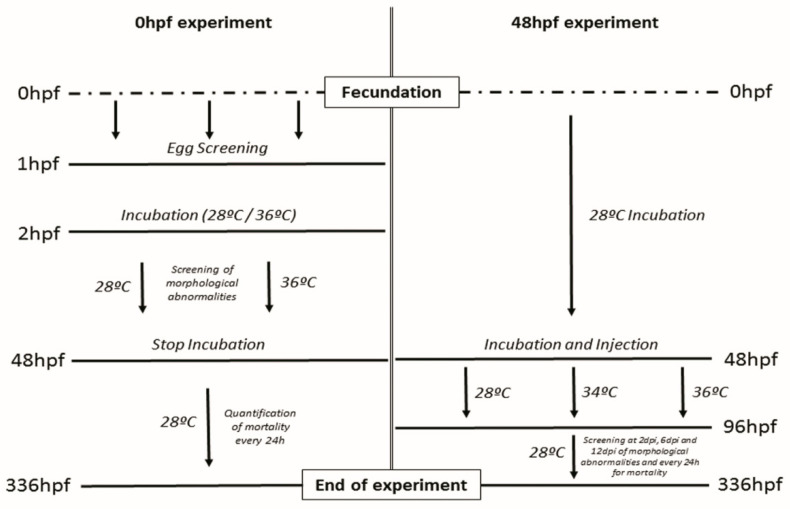
Overview of the two assays starting at two different start points. Hpf = hours post-fertilization. Dpi = days post-injection.

Embryos were collected at 0 hpf and screened after 1 hpf to guarantee a normal cell division, eggs showing abnormal or asymmetric cell division were discarded and replaced by normal ones in order to reach the selected number of embryos for each treatment. Embryos were placed before 2 hpf in Petri dishes at two different temperatures (28 °C and 36 °C) and incubated for 48 hpf screening the embryos for different malformations and mortality at previously determined critical developmental time points (5 h, 10 h, 24 h, 48 h) [15]. After incubation, the hatching rate was quantified, and the embryos were placed at 28 °C to assay the mortality at different time points up to 336 hpf (14 dpf) (Figure 1).

#### 2.4.2. Assay Starting at 48 hpf

Zebrafish embryos were incubated at 28 °C until 48 hpf. Afterward, embryos showing a normal developmental pattern were divided into three groups: (1) non-injected controls, (2) Injected with vehicle (RPMI) and (3) Injected with MCF7 breast cancer cell line. Embryos were then incubated for 48 h at 28 °C, 34 °C, and 36 °C until 4dpf. After differential temperature incubation, embryos were returned at 28 °C and were further incubated up to 14 dpf. Embryos were screened for mortality on a daily basis, and morphological abnormalities were screened at 0, 2, 6, and 12 days post-injection (dpi) (Figure 1).

### 2.5. Embryo Imaging

Embryos were screened using an AZ-100 (Nikon) fluorescence stereomicroscope to identify and quantify relevant abnormalities: spinal deviation, edema, hatching rate, and head deformation (including ear malformations, abnormal eye size and mouth) for all the treatments described in the previous section. Malformations are expressed as percentages of the total embryos alive at the time point analyzed.

### 2.6. RNA Isolation, cDNA Synthesis and qPCR

To determine the differential effect of temperature on gene expression at 34 °C and 36 °C, we selected genes previously shown to be affected by the temperature at 34 °C [10]. We also analyzed the expression of the following genes relevant for metabolic pathways that could be interfering with the normal proliferation and behavior of the xenografted-cells as a consequence of the cell–host interaction: development (*lft2*, *mmp9*, *haus3*, *junb-a* and *lum*), immune response (*socs3a*, *junb-a, tnfa*, *csf3a*, *csf3b and il1b*), stress response (*apex1*, *hspa9*), and metabolism (*per2*, *wisp*) (Table S1). For gene expression quantification, conditions from the previous experiment were replicated, dividing the embryos into three different groups based on incubation temperature (28 °C, 34 °C and 36 °C) starting at 48 hpf and up to 96 hpf. After differential temperature incubation, embryos were returned to 28 °C to recover for another 72 h (from 96 hpf to 168 hpf). A total of 10 embryos were euthanized and disaggregated in triplicate for each incubation temperature (28 °C, 34 °C and 36 °C) after 96 hpf (differential temperature effect) and after 168 hpf (recovery effect). We used the RNeasy mini kit (QIAGEN, Hilden, Germany) to extract embryo RNA from the cell suspension after disaggregation of each condition following the manufacturer’s instructions. After DNAse treatment, cDNA was synthesized using the AffinityScript multi-temperature cDNA synthesis kit (Agilent Technologies, Santa Clara, CA, USA), following the manufacturer’s instructions. Gene expression was assayed using the Brilliant III Ultra-Fast SYBR^®^ Green QPCR Master Mix (Agilent Technologies—Santa Clara, CA, USA) in a Stratagene Mx3005P quantitative PCR. Relative fold changes of gene expression normalized to β-actin as the housekeeping gene were calculated using the ∆∆Ct method.

### 2.7. Statistical Analysis

GraphPad Prism version 7.00 for Windows (GraphPad Software, La Jolla, CA, USA) was used for statistical analysis. Unpaired Student’s t-test was performed to analyze the mortality and morphological defects from 0 hpf to 48 hpf; differences were considered significant when *p* < 0.05. One-way ANOVA was used to analyze the morphological abnormalities experiment beginning at 48 h and the qPCR experiments. Differences were considered significant when *p* < 0.05.

## 3. Results

### 3.1. Mortality and Morphological Effects Comparison between 28 °C and 36 °C from 0 hpf to 48 hpf

We explored the possibility of exposing the embryos from nearly 0 hpf up to 48 hpf to a high incubation temperature (36 °C) and determine if this acclimation before the injection could interfere in the development and mortality of the embryos in the long-term.

Zebrafish embryos were harvested at 0 hpf, incubated at 28 °C and 36 °C for 48 h. Embryos incubated at 36 °C were returned to 28 °C, and incubation continued until 336 hpf (14 dpf). Different morphological abnormalities were observed at 36 °C, not evident at 28 °C. These included spinal deviation, probably due to a higher developmental speed at 36 °C, edema, and head deformation. Besides these, the hatching rate of the embryos was increased drastically at 36 °C (Figure S1). No statistical difference in mortality was observed for the embryos incubated up to 48 hpf at 28 °C compared to embryos incubated at 36 °C. Although the mortality of the embryos stabilizes from 24 hpf onwards, in the first 24 h of development, the mortality at 36 °C occurs earlier than at 28 °C (Figure S2B). To test if the incubation at 36 °C from 0 hpf to 48 hpf produce long term effects, the embryos were incubated at 28 °C for the rest of the experiment until 336 hpf (14 dpf), checking the mortality on a daily basis. Results show that there were no differences and, therefore, no long-term effects from the incubation at 36 °C in the first 48 hpf (Figure S2A).

### 3.2. Mortality and Morphological Analysis at Different Incubation Temperatures in 48 hpf Embryos

#### 3.2.1. Mortality

**Figure 2:**
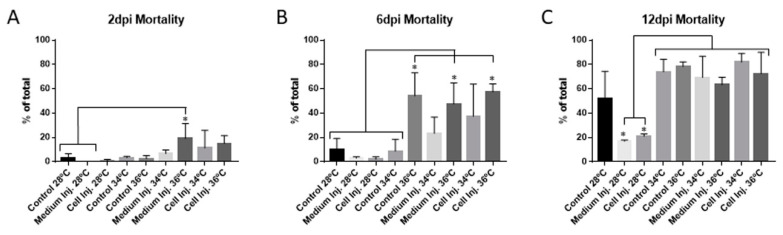
Mortality quantification at different time points comparing the different incubation conditions of the zebrafish embryos. (**A**) Mortality quantified at 2 dpi. (**B**) Mortality quantified at 6 dpi. (**C**) Mortality quantified at 12 dpi. Parameters are expressed as percentages from the total number of embryos assayed for each condition, comparing the incubation of the embryos from 2 dpi to 12 dpi at 28 °C, 34 °C and 36 °C. Error bars represent the standard deviation (SD). One-way ANOVA was performed, and differences were considered significant when * *p* < 0.05 (n_28 °C_ = 150, n_34 °C_ = 150, n_36 °C_ = 120). The experiment has been performed in triplicate.

Forty-eight hpf embryos were incubated at 28 °C, 34 °C, and 36 °C for 48 h. Mortality assessment showed a mild increase in mortality at 34 °C and 36 °C when compared to embryos incubated at 28 °C (Figure 2A). No differences were observed between injected embryos at 34 °C and 36 °C. These results imply that xenotransplantation assays are possible using 48 hpf embryos, at 36 °C for 48 h, without higher mortality in comparison to incubation at 34 °C.

Embryos incubated at 34 °C and 36 °C for 48 h were then returned to 28 °C, and incubation was carried on up to 6dpi. At this time, the mortality was significantly different between control, medium injected, and cell injected embryos at 36 °C compared to all the conditions at 28 °C and the controls incubated at 34 °C (Figure 2B). While no significant increase in mortality was observed at 34 °C, a clear tendency of higher mortality was observed that was later confirmed, after another six days of recovery at 28 °C. The mortality of the embryos incubated at 34 °C and 36 °C increased to around 80%, with significant differences between all the conditions assayed at these temperatures compared to those at 28 °C. The exception was 28 °C incubated controls due to the high variability obtained in this condition (Figure 2C). At this time point, at least 20% of the mortality can be explained by the undergoing metamorphosis process between 12 dpf and 14 dpf. Added to this is the mortality attributed to the high incubation temperatures and/or injection suffered during the experiment.

#### 3.2.2. Morphological Abnormalities

Representative morphological defects (Figure S1) were quantified and analyzed for the same experimental conditions described in the previous section, including xenografted embryos, even if the cells suffer from cell death during the process of returning the embryos to a normal temperature of 28 °C (Figure S3).

**Figure 3:**
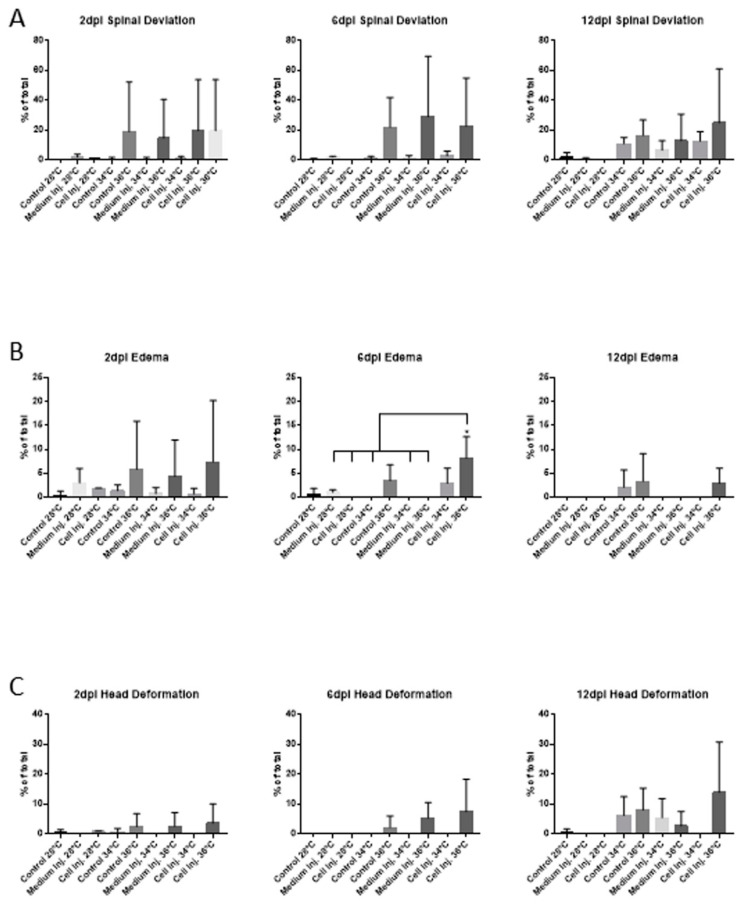
Morphological abnormalities quantification comparing the different times and incubation temperatures assayed. (**A**) Spinal deviation. (**B**) Edema. (**C**) Head deformation. In all cases, values are expressed as the percentage of the total number of embryos assayed for each condition comparing the incubation of the embryos for 48 h at 28 °C, 34 °C and 36 °C and the posterior recovery at 28 °C until the end of the experiment. Error bars represent the standard deviation (SD). One-way ANOVA was performed, and differences were considered significant when * *p* < 0.05 (n_28°C_ = 150, n_34°C_ = 150, n_36°C_ = 120). The experiment was performed in triplicate.

No clear differences in morphological abnormalities were observed after 2 dpi, 6 dpi or 12 dpi at 28 °C, 34 °C, and 36 °C except for the case edema for which a significant increase was observed in xenografted embryos incubated at 36 °C after 6 dpi (Figure 3). Therefore, related to xenograft assays, there are no statistical differences between cell injection at 34 °C and 36 °C in terms of morphological abnormalities of the embryos at 2 dpi. Even without statistical significance, spinal deviation and edema at 2 dpi is present in conditions related to the incubation at 36 °C (Figure 3A,B) and could be considered a teratogenic effect of the temperature (36 °C) at the final point of the xenograft experiment, but not interfering in mortality (Figure 2) or the correct proliferation of the injected cells, as described in previous studies [7].

#### 3.2.3. Gene Expression Related Changes

In order to address the metabolic pathways affected in embryos exposed to 34 °C and 36 °C, we measured gene expression changes of selected genes at different time points:

Gene expression after 48 h at 28 °C, 34 °C, and 36 °C.

**Figure 4:**
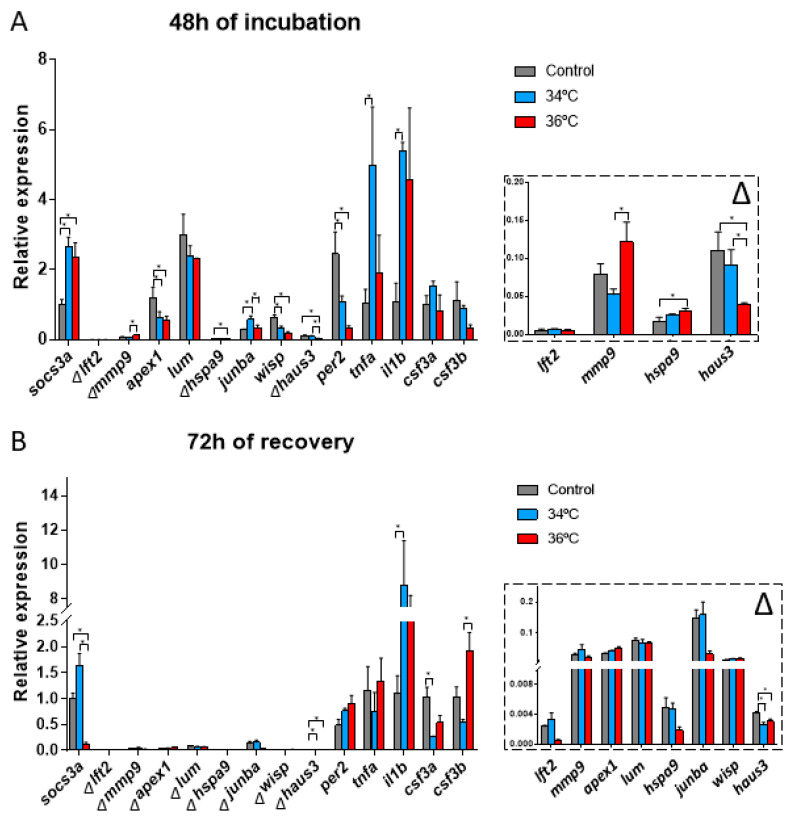
Embryo gene expression analyzed by qPCR after incubation at 28 °C, 34 °C, and 36 °C for 48 h (**A**) and for 48 h plus 72 h recovery at 28 °C (**B**). N = 10 embryos/temperature and time point in triplicate. One-way ANOVA was performed, and differences were considered significant when * *p* < 0.05. ∆ = in the detailed view of marked genes.

The expression of *lft2*, *lum*, *csf3a* and *csf3b* showed no significant differences between the temperatures assayed. On the contrary, *socs3a*, *mmp9*, *apex1, hspa9*, *junba*, *wisp*, *haus3*, *per2*, *tnfa* and *il1b* were up or downregulated at 34 °C and 36 °C compared to the controls at 28 °C (Figure 4A).

Differences in gene expression between 34 °C and 36 °C were observed for *mmp9*, *junba* and *haus3*. These genes are related to the developmental processes of the embryos, suggesting an altered development between the two incubation temperatures (Figure 4A) and also between these temperatures and that optimal for growth.

#### 3.2.4. Gene Expression after 72 h of Recovery at 28 °C

After the recovery for 72 h following the incubation at 34 °C and 36 °C for 48 h, some differences persist in the gene expression of the genes analyzed. The expression of *socs3a*, *haus3*, *il1b*, *csf3a* and *csf3b* were up or downregulated compared to the control at 28 °C (Figure 4B). Differences in gene expression in embryos incubated at 34 °C and 36 °C were also observed for *socs3a* and *csf3b*, both of them related to the immune response (Figure 4B). In the case of *socs3a,* an inverse response was observed for both conditions when compared to controls, while *csf3b* increased for embryos incubated at 36 °C after recovery at 28 °C with no such response observed for embryos incubated at 34 °C.

## 4. Discussion

Zebrafish has gained relevance for being an excellent animal model to study human cancer [21,22]. It is a low-cost, fast and accurate model for high-throughput screening of drugs or new compounds due to their mass offspring and the possibility of performing xenotransplantation assays [23,24]. This last has greatly evolved during the last decade with several standard protocols already established. Despite this, several drawbacks still remain [15], among which host integrity—due to the high incubation temperature needed for xenografted cells to grow properly—is key to obtain accurate results for tumor cell response to applied treatments. This is highlighted in a recent work in which the effect of 5-Fluorouracil in zebrafish xenografted colorectal cancer cells was assayed. Different inhibition ratios were obtained depending on the incubation temperature of the embryos, showing a stronger decrease in cancer cells when the incubation temperature was 36 °C compared to 34 °C [7].

We have previously shown that incubating embryos at 36 °C for up to three days is feasible, and no higher mortality is observed when compared to embryos incubated a 34 °C [7]. At the same time, it was recently reported that incubation of zebrafish embryos at 32.5 °C and above causes malformations [17]; other authors reported normal development up to 35.5 °C [25,26]. In this work, we aimed to finely study the effect of the temperature range used in xenograft experiments on the host and further explore the possibility of increasing the temperature of xenograft assays up to 36 °C.

In the first experiment, embryos were incubated at 36 °C from 0 hpf to 48 hpf to confirm if they could be more sensitive in this stage of development to temperature. While mortality was not different at 48 hpf when compared to previous reports [17], significant spinal deviation and edemas were present in most of the embryos incubated at 36 °C. Apart from that, at 48hpf, the hatching rate of the embryos was higher at 36 °C due to the increase in development speed possibly produced by temperature. Interestingly, the hatching rate of 36.5 °C is 0% [17], pointing to an inflexion point in the tolerated temperature between 36 °C and 36.5 °C. When fish were returned to an optimal temperature, after incubation at 34 °C and 36 °C, and incubated up to 14 dpi, no differences in mortality were observed between treatments at 28 °C, 34 °C, and 36 °C (Figure S2B). The zebrafish developmental stage most sensitive to temperature appears to be between 5 hpf and 24 hpf (Figure S2A).

In a second experiment, we incubated xenografted and control embryos for 2 days at 34 °C (the most used temperature for this type of experiment [27,28,29]) and 36 °C to compare temperature-induced mortality, morphological defects, and metabolism. In terms of mortality, the first stage of development from 0 hpf to 48 hpf (previous experiment) produced different results than those observed for the incubation at 36 °C from 48 hpf to 96 hpf, with the posterior recovery of the embryos from 96 hpf to 336 hpf. The different conditions assayed (control, injected with RPMI medium or injected with MCF7 cells) at this stage of development showed that the embryos are less sensitive to the higher temperature than in the first 48 h of development. The temperature has a higher impact on development between 0 hpf and 48 hpf, yielding more abnormalities and mortality of the embryos. The lack of differences in mortality and malformations in injected embryos between 34 °C and 36 °C after 2 dpi, from 48 hpf to 96 hpf, allow xenograft assays to be performed at 36 °C to determine xenografted cells proliferation, invasion or drug effects. To reach an incubation temperature ideal for tumor cells, without increased mortality or defects in embryos, a reduction in incubation time from 6 days to 2 days is required. Even this, more consistent and realistic results with regard to tumor cell proliferation could be expected. On the contrary, xenograft experiments at lower temperatures, with an increased incubation time, could lead to lower tumor cell proliferation and, consequently, an overestimation of the chemotherapeutic effect for assayed drugs [7].

We also analyzed the expression of genes related to development, immune system, stress, and metabolism to assess the effect of the incubation temperatures assayed over important metabolic pathways that could be influencing xenograft assays on zebrafish embryos according to previous works [10].

After 48 h of embryo incubation at 34 °C and 36 °C compared to the control at 28 °C, some differences in the gene expression were found. When compared to embryos incubated at 28 °C, the expression of *socs3a*, *hspa9*, *junba, tnfa* and *il1b* were upregulated, and the expression of *apex1*, *wisp*, *haus3* and *per2* were downregulated in embryos incubated at 34 °C and 36 °C. The most relevant differences observed in the present study are the ones that arose from the comparison of 34 °C against 36 °C (Figure 4A). Comparing results obtained at 28 °C with those obtained at 34 °C and 36 °C, *per2*, a gene which downregulation enhances the expression of proinflammatory cytokines [30], was one of the most downregulated genes due to the temperature increase. On the contrary, *socs3a*, involved in immune response and inflammatory pathways, tissue regeneration and in the repression of the STAT3 signaling pathway, was upregulated. The STAT3 pathway is involved in the regeneration of the liver, skin, fin, retinas, and the sensory epithelium cells of the inner ear of zebrafish embryos, apart from being involved in cell proliferation, migration, and survival [31,32]. A differential gene expression of *mmp9*, *junba* and *haus3*, genes involved in the development, was observed between 34 °C and 36 °C. Even that, the relative expression changes of these last-mentioned genes are really low (Figure 4A). This result supports selecting 36 °C as a good temperature for xenograft assays when the incubation time is reduced.

After embryo recovery at 28 °C for 72 h, gene expression reverted to normal (gene expression observed in embryos continuously incubated at 28 °C). Even this, some differences still persisted for *socs3a*, *haus3* and *csf3b* (Figure 4B). The expression of *socs3a* was downregulated after recovery in embryos incubated at 36 °C when compared to embryos incubated at 34 °C. The tissue regeneration during recovery at 28 °C could be the reason behind this observation. On the other hand, *csf3b*, involved in immune response (neutrophil chemotaxis), remains upregulated after recovery from 36 °C compared to recovery from incubation at 34 °C. This could be suggesting that a persistent inflammation due to the stress induced by the high incubation temperature could be a long-term effect. All-in-all, most of the alterations observed in gene expression consequence of incubation for 48 h at temperatures higher than optimal up to 36 °C, mostly revert to normal after a recovery period of 72 h.

## 5. Conclusions

While there are differences in mortality and certain malformations are observed in zebrafish embryos incubated at 34 °C and 36 °C from 0 hpf to 48 hpf, this is not the case when embryos are incubated at the same temperatures, but from 48 hpf to 96 hpf. This implies that a 2 day incubation period from 48 hpf to 96 hpf at 36 °C could be used for xenograft assays in the yolk sack or other parts of embryos for more robust and accurate results in terms of tumor cell proliferation and/or invasion in response to drug testing in vivo.

The temperature effects over gene expression were mostly observed between the control condition at 28 °C with respect to 34 °C/36 °C after 48 h of incubation. After the 72 h recovery period, the temperature effects over gene expression is reverted for most genes, with the exception of *socs3a*, *haus3*, *il1b*, *csf3a* and *csf3b.* Differences in gene expression for embryos incubated at 34 °C and 36 °C were observed for genes related to development (*mmp9*, *junba* and *haus3*), not interfering in the xenograft technique.

## Data Availability

The data presented in this study are available within the article and/or Supplementary Materials.

## Supplementary Materials

The following are available online at https://www.mdpi.com/2073-4425/12/1/113/s1, Figure S1: Effect of temperature on development from 0hpf to 48hpf in zebrafish embryos. Figure S2: Effect of temperature on the viability of 48hpf zebrafish embryos. Figure S3: Representative images of each time point of the xenografted embryos with MCF7-GFP human cells. Table S1: qPCR primers sequence.
