# Supplementary material for: Morphological Abnormalities and Gene Expression Changes Caused by High Incubation Temperatures in Zebrafish Xenografts with Human Cancer Cells
Source: Genes (Basel). 2021 Jan 19;12(1):113. doi: 10.3390/genes12010113 (PMC7832305; doi:10.3390/genes12010113)
Supplement: Supplementary file 1 [file genes-12-00113-s001.zip › Reviewer_answers.docx]

**REVIEWER 1**

Suggested Improvements

1) *I don’t understand the relevance of figures 2 and 3 to the overall aim. The embryos in these figures are not xenografted and generally show temperature effects that are well established in other studies. It is already known that growing embryos at temperatures greater than 28oC from 0 hpf will be teratogenic. Most, if not all, xenograft studies use embryos grown at 28oC from 0-48 hpf so the relevance of this data is not clear. I suggest this data is moved to a supplemental section.*

**-According to the suggestions of Reviewer 1 and 2, the information related to the experiment ranging from 0hpf to 48hpf was moved to supplementary data.**

2) *Figures 3, 4 and 5. Given that most xenografts are studied within the first 2-4 days of injection I do not understand the relevance of the 6 dpi and more especially the 12 dpi timepoints included in these figures.*

**-Part of the literature stablish that xenograft assays are performed at 34ºC and 6 days as a standard protocol, being that the reason for the 6dpi time point. The 12dpi time point was selected to check long-term effects of this incubation temperature over the host, especially during the metamorphosis, regardless if the xenograft assays are not kept until that timepoint.**

3) *Figure 2 – the arrows highlighting the defects in panel B are too small to see. In addition, this figure would be improved by subpanel callouts so the exact defect can be related to each photo.*

**-The Figure 2 (now Figure S1) has been improved by adding bigger arrows and subpanels with the exact defect. Apart from that, we changed the orientation of the images in order to be more clear and homogeneous.**

4) *Figure 2 – Hatching rate. The authors measure hatching rate in Fig 2A. How was this determined as all embryos will hatch given enough time. Presumably this was measured at 48 hpf? This detail should be included in the figure and methods.*

**-We added a statement in the Figure 2 (now S1) legend to clarify that the quantification of the malformations and hatching rate were performed at 48hpf. Apart from that, we included in the text, in Methods section (‘Assay starting at 0hpf’) stating that hatching rate was quantified at 48hpf.**

5) *Figure 3. Panels A and B are the wrong way around.*

**-Panels A and B in Figure 3 (now Figure S2) have been corrected.**

6) All graphs. The identity of the error bars should be stated in the figure legends for all graphs.

**-The identity of the error bars is now stated for each figure in each legend.**

7) *Experiment for Figure 4-5. This is the key experiment in the paper. Unfortunately, it is missing a control – xenografted embryos at 28oC. The only control used is un-injected embryos at 28oC. Because of this, the authors have really only compared the temperature effects between 34 and 36oC and unsurprisingly find little difference. I believe this data would be more meaningful if it also included the controls stated below.*

*-28oC medium-injected embryos*

*-28oC xenografted embryos*

*The authors themselves acknowledge that they are unable to distinguish between the temperature and xenografting effects in lines 230-236. Related to this, comparing the significance of xenografted 36oC embryos to un-injected 28oC embryos in Figure 5B (6 dpi oedema) is not a relevant comparison.*

**-The control experiment suggested have been performed and included in the Figure 5 (now Figure 3), and new text for these results have been included in the manuscript.**

8) Figure 6. It is not clear what the fold-change is compared to. Presumably 28oC? This should be added to the Y axis of both graphs and clarified in the legend.

**-The Figure 6 (now Figure 4) has been revised to clarify the comparisons of each gene and all the significances have been added.**

9) Figure 6. It is not clear what significance values for per2 and socs3a actually relate to? Is it a significant difference between the fold change at 34 and 36oC or for 34 to 28oC or 36 to 28oC or both 34/36 and 28oC? This needs to be clarified.

**-This has been addressed in the previous point rebuilding the Figure.**

10) *There are a number of instances where the authors presume significance where the data does not support this. They use words such as “there is a tendency” or that genes are “down regulated” when their data shows no significant difference and therefore it should be stated that there is no change.*

*-Examples are:*

*Line 204-205 “Nevertheless an increased mortality tendancy was observed...” There is no significant difference in mortality from Fig 4A. This line should be removed.*

*Line 218-219 “part of this mortality ..due to the changes related with incubation tempature or injection”. The data in fig 4C does not show a significant difference between the different temperatures or with/without xenografting and this line should be removed.*

*Line 239 “..tendency for spinal deviation” This data is not significant and this line should be removed.*

*Lines 263-276. Only Per2 (at 48h) and Socs3a (at 72h) show any significant differences. Mention of any other genes being up or down regulated should be removed or the significance values added to the graph.*

**-The manuscript has been revised in order to correct these assumptions.**

11) As the gene expression changes are referring to mRNA, the authors should use lowercase italics for each gene investigated and also in the subsequent discussion as per standard zebrafish nomenclature. For example: per2 not Per2.

**-The name of the genes has been updated according to the zebrafish nomenclature.**

12) The authors mention the possibility that temperature could alter the expression of zebrafish pro-inflammatory cytokines, yet strangely their expression analysis did not include any of these. For example, they could have included: tnfa, il1b, gcsf all genes commonly examined in the zebrafish model.

**-A qPCR experiment analysing the suggested genes is now included in the manuscript, and commented in the results and discussion sections.**

13) The authors refer to a 3dpi timepoint at line 213 and a 72h timepoint at line 203. These timepoints do not match the data described. I think 72h is actually 48h and the 3 dpi is 2 dpi?

**-The time points in lines 203 and 213 have been corrected.**

**REVIEWER 2**

*In addition, embryo survival and developmental defects following short-term (48 h) exposure of embryos just after fertilization was evaluated. The purpose of this early exposure experiment is less clear. While I see no reason why this data should not be published, these results seem disconnected from the main topic of the manuscript. It is my understanding that this served as a demonstration that the embryos are more sensitive to elevated temperatures early in the development than at the age when most xenograft experiments are performed. Perhaps the rationale behind this should be clearly explained.*

**-According to the suggestions of reviewer 1 and 2, the information regarding the experiment from 0hpf to 48hpf was moved to supplementary data.**

There are some minor issues with the overall text:

*Language: I think it is crucial that the authors have the language and punctuation of the manuscript checked. I restrained myself from commenting on individual errors. Firstly, there are too many; secondly, I don't believe I can recognize all of them and suggest appropriate changes. Some of these errors are just typos or issues with punctuation (I suggest reading the MDPI guidelines on English editing, which are available online), so they should be easy to find when the authors proofread the manuscript carefully. However, there are also serious grammatical issues. I thus hope that the authors will consider consulting a native speaker or at least someone with a passion for English to help improve the text.*

**-Manuscript has been proofread for grammatical and punctuation errors.**

*Explanations of the methods are scattered throughout the manuscript – from the Introduction to the Discussion. They should be provided in the Methods. I suggest that the authors collect the parts of text describing methodology (sample sizes, genes analysed, determination of hatching rates, duration of different incubations etc.) and move them to the appropriate section. Furthermore, some information concerning methodology should be added (I pointed out such instances in the specific comments).*

**-As suggested by the reviewer, we revised the methodological information and included it in the methods section.**

*The authors used Petri dishes with 50 embryos per dish in their incubations. If a few embryos in a petri dish die or are stressed, this will affect the other embryos sharing the same petri dish, even if the medium is changed every 24 h. These embryos thus do not represent independent measurements, while individual Petri dishes do. Considering that the authors approached statistical analysis by using repeats as independent measurements, their approach to incubation is, in my opinion, legitimate for this type of analysis. However, sample sizes must be provided, listing the number of repeats or the total number of petri dishes per treatment (which is what the statistics were performed on), not the total numbers of embryos in the study. The captions to Figs. 2 and 3 claim the statistical datasets are 270 and 700, but these are likely the numbers of embryos. I find it hard to imagine the authors repeated the experiment 700 times.*

**-We clarified in the section ‘Incubation and assay conditions’ in Methods that at least two plates per replica were used containing 50 embryos per plate.**

*I think it would be better to incubate the embryos in multi-well plates, where each embryo would have its own well and would thus represent an independent measurement. This would then allow frequencies of malformation to be analyzed with variations of the Chi-square test, which would be a better approach to determine differences in frequencies.*

**-We agree with this statement. It would be a better approach to use the 24-well plates in order to avoid any interferences that could arise from bulk incubation in the Petri dish. We will consider this valuable suggestion for our experiments to come.**

*All laboratory models, in vitro or in vivo, have their limitations. Considering that the yolk sac of a zebrafish is a very different environment from what human cells normally encounter, the difference in temperature might not be the only factor worth addressing. While the suggested incubation temperature might be important for certain processes in some cell lines, this might not be the case in experiments that use a different approach (such as injection in the circulation or the brain) or other types of cancer (such as melanoma). Therefore, I can imagine that there might be experiments in which the higher incubation temperature suggested by the authors is not as beneficial as in the case of MCF7 cell proliferation in the yolk sac. My point is that the authors might want to limit their conclusions to the specific type of experiment they performed, which is quantification of cancer cell proliferation in the yolk sack.*

**-The conclusions have been re-written in order to limit them to the yolk. Even that, the temperature in zebrafish xenograft assays of any type is quite important for cancer cells growth, at least to our knowledge, as long as all the cell lines have an optimal incubation temperature of 37ºC *in vitro*.**

Specific comments:

-Title

*I suggest »metabolism-related changes«, with a hyphen. Alternatively, this could be replaced with something more specific. As a reader, I don't clearly envision what the manuscript is about when I read »metabolism-related changes«. Perhaps replacing this with »changes in gene expression« or something even more specific would be an improvement in clarity.*

*What I am missing in the title at first glance is information that the manuscript is about cancer. Xenografted embryos can mean anything.*

*Perhaps »Quantification of« can be left out to make the title shorter.*

**-The title has been changed as suggested.**

-Keywords

*The keywords chosen are fine, but they are abundantly used in the title and abstract already. I suggest that the authors consider using keywords that would add to what is already present in the abstract.*

**-The keywords have been changed to better identify the paper with other words and synonyms.**

-Introduction

*34 »This involves the injection of human cancer cells inside the yolk or in the circulation of the zebrafish embryos at 48 hours post fertilization«: I suggest the authors expand their literature overview to include studies in which cancer cells were xenografted elsewhere. The yolk and circulation are common enough, but they are not the only sites of injection, nor has this only been done at 48 hpf.*

**-It is true that depending on the aim of the study, there are other sites of injection, and even experiments with adult fish can be carried out. The introduction was changed to clarify this point, and a reference of an actual review related to sites and time points of injections was added.**

*39 »the incubation temperature of the embryos have not received much attention« – This is not entirely true. There are quite some studies that examined the effects of incubation temperature, which the authors themselves list a few paragraphs further and which the authors themselves published!*

**-We stated this because compared to the works published regarding the optimization of other conditions (site of injection, image analysis…) not a lot of researchers focused on the temperature upon the cells or the host.**

*63 »While most of the xenograft studies in the literature perform the incubation at 34ºC…«: this claim requires some support. 28-33 degrees are quite common. There are papers that have already provided great tables summarizing the relevant methodology (such as the paper published by the same authors in BMC Cancer in 2018), so referring to such lists (or, perhaps, even expanding them with more recent publications?) would give a more accurate representation of what people in this field most often do and perhaps allow for better argumentation for why the study was performed in this way.*

**-We included a reference to our paper so the reader can check a list of common temperatures and included ‘*34ºC or less temperatures*’ to highlight the point that most studies do not use a temperature higher than 34ºC.**

*70 »temperature modifies metabolic pathways in the zebrafish embryos involved in immune/stress response, inflammation, metabolism ...«: I think it is self-evident that metabolic pathways are involved in metabolism.*

**-The phrase has been corrected.**

*78-89: »we selected genes ...«: This belongs to the methods section (where the authors can also refer to the supplementary material, which is currently not mentioned in text).*

**-The statement in the Introduction section has been moved to the Methods section (RNA isolation, cDNA synthesis and qPCR), mentioning the Supplementary Table 1.**

-Methods

*101 Cell injection in zebrafish embryos: Approximately how many cells (on average) were injected per embryo?*

**-The average of cells injected into the embryo using MCF7-GFP cell line is now stated in Methods section.**

*108 Incubation and assays conditions: How many Petri dishes were used per condition? The experiments were performed in triplicate, this is stated. Was one Petri dish per treatment used in each replicate or were there several? In other words, what is the sample size?*

**-We clarified in the section ‘Incubation and assay conditions’ in Methods that at least two plates per replica were used containing 50 embryos per plate.**

*114 »Gemma 75 (search)«*

*I don't think »search« is the producer of Gemma.*

**-This error has been corrected stating the company.**

*146 RNA isolation, cDNA synthesis and qPCR*

*This would be a good place to refer to the Supplement and to explain which genes were studied.*

**-This has been done along with the last comment of the Introduction section and corrected.**

*154 »Relative fold changes of gene expression were calculated using the ΔΔCt method«: Which housekeeping genes were used for normalization?*

**-The housekeeping gene used for normalization has been added to Methods section.**

*156 Statistical analysis*

*Considering the numbers of pairwise comparisons (also in instances when only »ANOVA« is reported as the statistical method used), what type of correction was used for correct for the number of comparisons? (e.g. Bonferroni, Tukey, …)*

**-Tukey’s correction used to perform these statistical analyses has been added to the Statistical analysis section.**

*161 »two-way ANOVA was performed to analyze the qPCR experiments«: How and why did the authors use two-way ANOVA in this case? Please, explain what were the two independent factors considered and what were the results of this analysis? Is there evidence for an interaction between these factors?*

**-The statistical analysis was corrected and substituted by one-way anova to compare the different conditions (28ºC, 34ºC and 36ºC).**

-Results

*174 »the hatching rate of the embryos is increased drastically…«: What specifically does hatching rate mean? Percentage of embryos that have hatched by 48 hpf? This should be explained in the methods section.*

**-Now its stated in the Methods section that hatching rate was measured at 48hpf.**

*193 »Quantification and comparison of mortality and morphological effects...«: This paragraph does not belong in the Results.*

**-The paragraph has been removed from Results section and integrated into Methods section.**

*204 »an increased mortality tendency was observed in injected conditions (RPMI and MCF7 human cancer cells) at 34ºC and 36ºC, suggesting increased sensibility to temperature when the injection is performed…«*

*There are two reasons why this conclusion cannot be made. 1) Increased mortality, if it were observed in this case, could simply result from injection itself, not from resulting greater sensitivity to temperature. The reason behind it could not be determined unless there was also a group of injected embryos incubated at 28 degrees. 2) There are no statistically significant differences between control and treatment groups, so there is no reason to speak of increased mortality in any group.*

*By the way, comparing injected and non-injected groups at different temperatures would be a good example of an experiment where two-way ANOVA might be used.*

**-The text has been changed according to the new experiment including medium and cells injected controls incubated at 28ºC.**

*227 Morphological abnormalities of the embryos*

*This section comments on differences between different incubation temperatures and treatments. However, few of the observed changes were statistically significant. While the graphs suggest that elevated temperature causes greater incidence of malformations, either more experimental repeats or a different experimental design would be necessary to demonstrate this. Currently, there are very few cases (see Fig. 5) where such differences were demonstrated.*

**-We have clarified in which cases the differences are significant and reduced the statements were even with incidence, there is no significance to confirm that.**

*228 »Representative morphological defects represented in Fig. S1«: Fig. S1 does not show morphological defects.*

**-Now the Fig. S1 represents morphological defects.**

*254 »Gene expression quantification«: This text belongs to the Methods section.*

**-The paragraph now is integrated in the Methods section.**

*263-276 Gene expression*

*This segment is very hard for the reader to interpret.*

*It is unclear what the reported levels of significance refer to and what is compared. For example, Per2 is significantly differently expressed at 34 and 36 degrees, this can be made out, but which levels of expression differ significantly from control? The text leads the reader to believe that this was determined completely arbitrarily. For example, after 48 h, Junba or Haus3 expression are said to not be different from control, while Lum expression is said to be different. On what ground? Everything is even more confusing after 72 h, where it is stated that the expressions of almost all genes return to normal, but that is not evident from the figure – the only thing we can make out is that most genes are drastically downregulated in the 36 degrees group. In the case of Socs3a, there is an asterisk on the figure, but are these the only expression levels that differ between 34 and 36 or is this the only case where one (which one? or both?) of them differs from control?*

*In short, it is very important that the authors clarify what is compared with what. If necessary, provide additional figures to show differences between gene expression in control and treatment groups.*

*Please, also explain (here or in the Methods) that control embryos incubated at 28 degrees for 48 and 120 hours were the baseline to which expression levels at these two time-points were compared.*

**-We changed the Figure for gene expression (now Figure 4) to make clear that comparisons against the control at 28ºC as a baseline are the case, and to clarify the significance in each case.**

-Discussion

The title of this section, in accordance with the journal's guidelines, should be Discussion, not Discussions

**-The title has been corrected.**

*The issues of the Results apply to their interpretation in the Discussion as well. The text of the Discussion should therefore be modified, taking these issues into account.*

*320-324: The authors’ interpretation of the results is in conflict with their report of the results themselves. While they state that 36 degrees leads to more malformations, they then conclude that there are no differences and that this is the optimal temperature for incubation.*

**-We have corrected the Discussion section and the Conclusions and stated that even though there are malformations over time (6dpi and 12dpi), the results obtained at 2dpi, the xenograft assays time-window, are not significant between 34ºC and 36ºC.**

*304: »due to the increase in development speed«: This is a possibility, not a certainty.*

**-The statement has been corrected.**

*306 »pointing to an inflexion point in the tolerated temperature between 36ºC and 36’5ºC«: I don't think the study presented in this manuscript can truly be compared with reference 15, as the embryos in that study were continuously subjected to high temperature whereas in this study, they were only incubated at 36 degrees for 48 hours.*

**-The hatching rate at 36’5ºC in the reference (Pype et al., 2015) has been measured at 48hpf incubating the embryos from 2,5hpf at the mentioned temperature. In our study the incubation time was the same but instead of 36’5ºC it was performed at 36ºC.**

*326 »Otherwise, lower temperatures could lead to lower tumor cell proliferation, ending up in an overestimation of the chemotherapeutic effect for assayed drugs«: Why should that be? If the temperature affects proliferation, it should affect the control just as well as the treatment group.*

**-Temperature affects proliferation of the human cancer injected cells in vitro and in vivo. Most chemotherapeutic compounds target proliferative mechanisms of tumour cells. In cells proliferating below their optimal capacity consequence of the lower temperature, the active concentration of the chemotherapeutic agent could be miss calculated.**

*355 »Per2 with the relation with pro-inflammatory cytokines, is down-regulated to promote the inflammatory response«: If it is pro-inflammatory, why does its downregulation promote inflammation?*

**-This point has been clarified in the text Per2 promotes the expression of this cytokines when this gene is downregulated.**

-Conclusions

*The main conclusion stated by the authors is that 36 degrees should be the temperature of choice for this type of experiments. The first sentence of the conclusions is in accordance with this (although the results suggest that this might also be a type 2 statistical error, which should be clarified). However, the gene expression analysis is hardly in agreement with the main conclusion. Perhaps the conclusions are not this simple?*

**-The conclusions have been re-written to reflect the changes made to the whole manuscript.**

-Figures

Most figures show graphs, but the error bars are not interpreted in any of them. Do they show standard deviation or standard error?

**-This point has been clarified in the legends of all graphs.**

*Fig. 1 – hours post fecundation*

*Hpf is defined as »hours post fertilization« in the Introduction. I think fertilization is used much more often than fecundation, so I suggest that fertilization is used throughout the text and figures.*

**-This error has been corrected in Figure 1 legend.**

*Fig. 2 – it would be helpful if images of different malformations were labelled so that it would be clear to the reader which image represents which malformation. Furthermore, the change in magnification between the top two images and the bottom four images should be indicated, at least in the caption, so that it is clear which scale bar is valid for which images.*

*As far as head deformation goes, the embryo in the image also has edema and its head is not obviously different from the other embryo with edema. If head deformation is a morphological change independent of edema, provide an image clearly depicting only head deformation.*

**-Figure 2 have been improved by adding labels for each deformation, the scale bars have been clarified in the caption and in the image. To properly show the morphological defect, the head deformation image was changed.**

*Fig. 3 – I suspect A and B have been switched.*

**-The A and B where switched, it has been corrected.**

*Fig. 6 – I am missing a measure of variability. Other graphs have error bars, these should have them, too. Since the embryos were pooled three times, this is sufficient to provide some measure of variability between these repeats.*

**-The Figure 6 (now Figure 4) has been improved, and error bars were added.**

**REVIEWER 3**

*Authors claim that 36ºC is a better temperature for the xenograft assay- however the data they show suggest the opposite– and when describing/interpreting the data that they show they ignore their own results….*

*For example, the authors show:*

***-Figure 4****- 6dpi mortality- at 36ºC non-injected fish have significant higher mortality than at 28ºC or 34ºC and in xenografts (injected) there is a clear tendency to higher mortality at 36ºC – the variation is huge …-?*

***-Figure 5A****- 2dpi/6dpi- xenografts @34ºC no spinal deviation vs a clear spinal deviation of xenografts @36ºC*

***-Figure 5B****-2dpi/6dpi- xenografts @34ºC no edema vs a clear increase in edema of xenografts @36ºC*

***-Figure 5C****-2dpi/6dpi/12 xenografts @34ºC no head deformation vs head deformation at 36ºC*

*So do not understand when authors claim in Line 237 “Therefore, related to xenograft assays there are no differences between cell injection at 34oC and 36oC in terms of morphological abnormalities of the embryos at 2dpi.”???*

**-The conclusions have been re-written in order to be more precise about our results in terms of accuracy and interpretation. All the differences mentioned in Figures 4 and 5 (now Figures 2 and 3) are not statistically significant, and the statements of tendencies have been corrected according to that suggested by Reviewer 2.**

**MAJOR**

*1-Introduction- line 34- zebrafish xenografts can be generated by injecting in the yolk, in circulation or in the periviteline space (PVS). It has been extensively shown that human tumor cells can form efficient tumor masses in the PVS in contrast to the yolk injection where cells tend to die.*

*REF-for example:*

*•Zhao, C.; Wang, X.; Zhao, Y.; Li, Z.; Lin, S.; Wei, Y.; Yang, H. A novel xenograft model in zebrafish for high-resolution investigating dynamics of neovascularization in tumors. PLoS ONE 2011, 6, e21768.*

*•Fior,R.;Póvoa,V.;Mendes,R.V.;Carvalho,T.;Gomes,A.;Figueiredo,N.;Ferreira,M.G.Single-cellfunctional and chemosensitive profiling of combinatorial colorectal therapy in zebrafish xenografts. Natl. Acad. Sci. USA 2017, 114, E8234–E8243*

*•Chapman, A.; Fernandez del Ama, L.; Ferguson, J.; Kamarashev, J.; Wellbrock, C.; Hurlstone, A. Heterogeneous tumor subpopulations cooperate to drive invasion. Cell Rep. 2014, 8, 688–695.*

*•Costa, B.; Ferreira, S.; Povoa, V.; Cardoso, M.; Vieira, S.; Stroom, J.; Pares, O.; Rio-Tinto, R.; Fidalgo, P.; Figueiredo, N.; et al. Developments in Zebrafish avatars as radiotherapy sensitivity reporters – towards personalized medicine. EBiomedicine 2019, 16, 102578.*

*•RohJohnson,M.;Shah,A.N.;Stonick,J.A.;Poudel,K.R.;Kargl,J.;Yang,G.H.;diMartino,J.;Hernandez,R.E.; Gast, C.E.; Zarour, L.R.; et al. Macrophage-Dependent Cytoplasmic Transfer during Melanoma Invasion In Vivo. Dev Cell 2017, 43, 549–562.*

**-We have corrected the statement, added the perivitelline space among other sites of injection and two extra references as suggested by the reviewer.**

*2. Why not compare 28ºC with 34ºC and 36ºC from 0hpf?? Only 36ºC?? since the second batch of experiments aim to compare to 34ºC? In our experience if we incubate – rear embryos from 0hpf in 34ºC we do not observe major developmental effects and after injection fish have less edema…*

**-We performed this assay to check if the maximum temperature would affect the embryos at this developmental stage (before 48hpf) to have more information about this unusual temperature. There are more studies assaying 34ºC (and lower) from 0hpf to 48hpf so we did not consider to add that temperature to our experiments.**

*3. Figure 1- images should all be oriented according to the Anterior/posterior/ dorsal ventral axis – anterior to the left / posterior to the right / dorsal up / ventral down*.

**-Figure 1 (now Figure S1) has been improved to fit the standard orientation of the embryos.**

*4. line 171- 336hpf is confusing is better to indicate 14dpf.*

**- 14dpf now replaces each mention to 336h.**

*5. Not a single image representative of xenografts with MCF7 cells …should show the xenografts in the various conditions and time points*

**-The Figure S3 has been added to Supplementary data to represent the state of the cells at each time point.**

*6. Fig4- 6dpi mortality- at 36ºC non-injected fish have significant higher mortality than at 28ºC or 34ºC and in xenografts (injected) there is a clear tendency to higher mortality at 36ºC – the variation is huge …-? No N is specified. How many fish were analysed in the end?*

**-The N is now included (2 plates with 50 embryos each plate per replica / 3 replicas). Due to the high dispersion of the data we did not found statistical significance even if there is a tendency in the data.**

*7. Fig.4 - mortality at 12dpi is huge even in controls – suggesting a problem in rearing / feeding the larvae…almost all fish die?*

**-The conditions of the embryos were checked every day. The controls at 28ºC have a mortality of 50% that we attributed to the metamorphosis suffered by the larvae between 10-14dpf. In the other conditions the mortality was higher.**

*8. 12dpi- Are the xenografts viable- do they still have human tumor cells - it would be very important to show with clear images.*

**-At 12dpi the xenografts are not viable and all the cells dye since they are incubated at 28ºC from the end of the 2-day incubation at different temperatures until the end of the experiment (14 dpf).**

*9. Line 228 “Fig. S1“, could not find this figure – is probably fig 1?*

**-We have corrected this error.**

*10. Line 237 “Therefore, related to xenograft assays there are no differences between cell injection at 34oC and 36oC in terms of morphological abnormalities of the embryos at 2dpi.” Figure 5 shows exactly the opposite of this:*

*• Figure 5A- 2dpi/6dpi- xenografts @34ºC no spinal deviation vs a clear spinal deviation of xenografts @36ºC*

*• Figure 5B-2dpi/6dpi- xenografts @34ºC no edema vs a clear increase in edema of xenografts @36ºC*

*• Figure 5C-2dpi/6dpi/12 xenografts @34ºC no head deformation vs head deformation at 36ºC*

**-Figure 5 (now Figure 3) shows what we stated in the line 237. In 5A, 5B and 5C, even with a tendency, there is no statistical significance. The variation between samples is big even with a big number of samples per replica.**

*10. Figure 6 – gene expression – no error bars – 10 embryos per replicate seems very small number …and is clear that 36ºC once more impacts much more on the physiology of the fish than 34º …*

**-We have corrected the Figure 6 (now Figure 4) with the error bars and improved the figure to be more easy to understand. The number of embryos were pools of 10 per replica with 3 biological replicates in the whole experiment, this information was included in the methods section.**

*11. All interpretation is biased to support a previous publication – which unfortunately in my opinion does not show what they claim – GFP quantification of cells injected in the yolk cannot account for proliferation…need to do confocal imaging with PHH3 marker (or EdU/BrdU) and quantify number of cells undergoing active proliferation and count number of cells – images shown in BMC-Cancer are not convincing, in our experience injecting in the PVS yields much better results with human cells clearly undergoing mitosis and proliferation whereas injection in the yolk leads generally to cell death.*

**-We have corrected the interpretation according to new experiments and results, being more critic with the data obtained.**

*12. The conclusions are not in accordance with the data that the authors present – once more they finish their paper with not only over-interpretation but also what seems a clear bias....*

**-The conclusions were re-written.**
