# Supplementary figures and images for: Morphological Abnormalities and Gene Expression Changes Caused by High Incubation Temperatures in Zebrafish Xenografts with Human Cancer Cells
Source: Genes (Basel). 2021 Jan 19;12(1):113. doi: 10.3390/genes12010113 (PMC7832305; doi:10.3390/genes12010113)

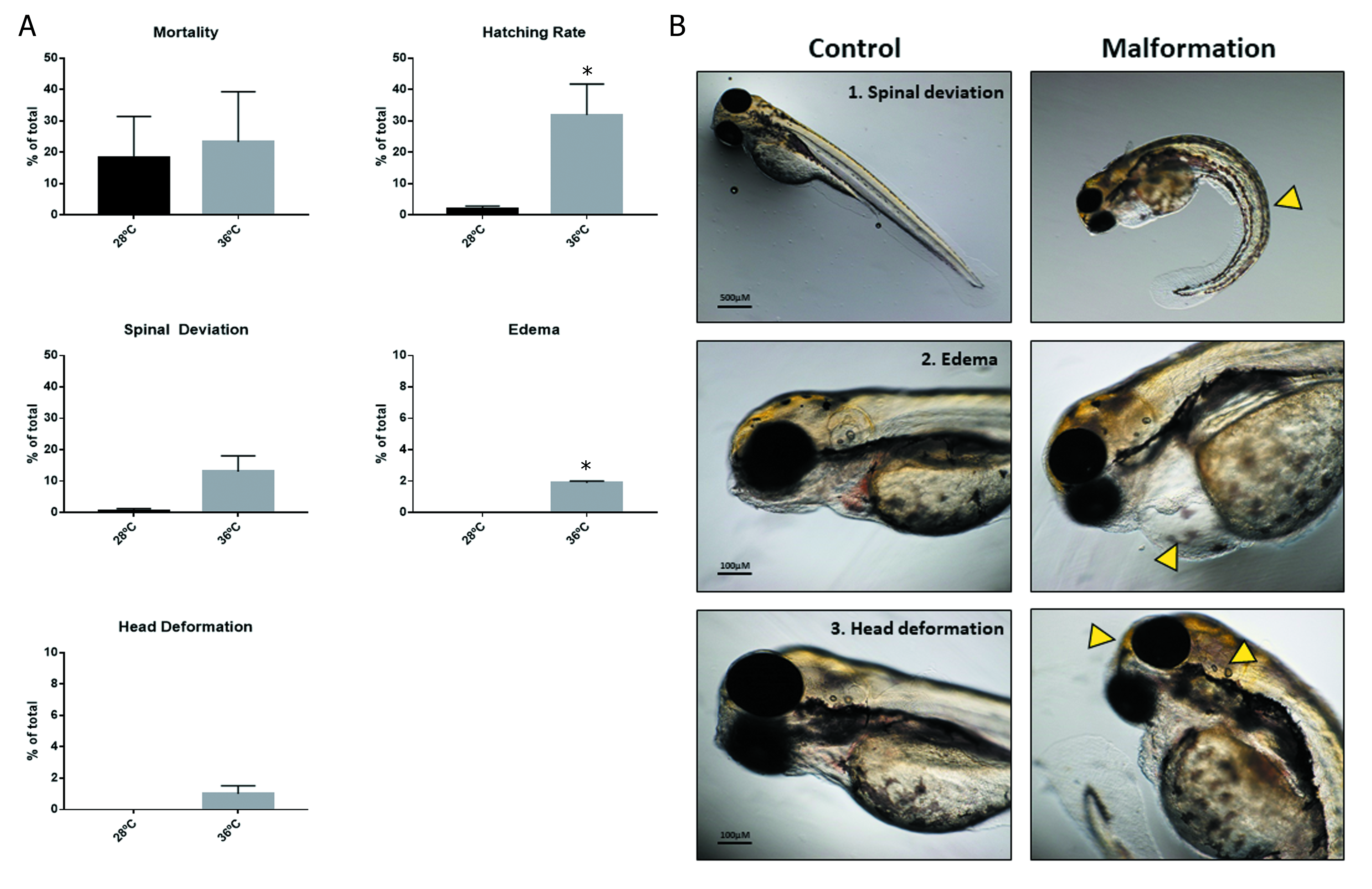

Supplement: Supplementary file 1 [file genes-12-00113-s001.zip › Cabezas-SainzFigS1.tif]

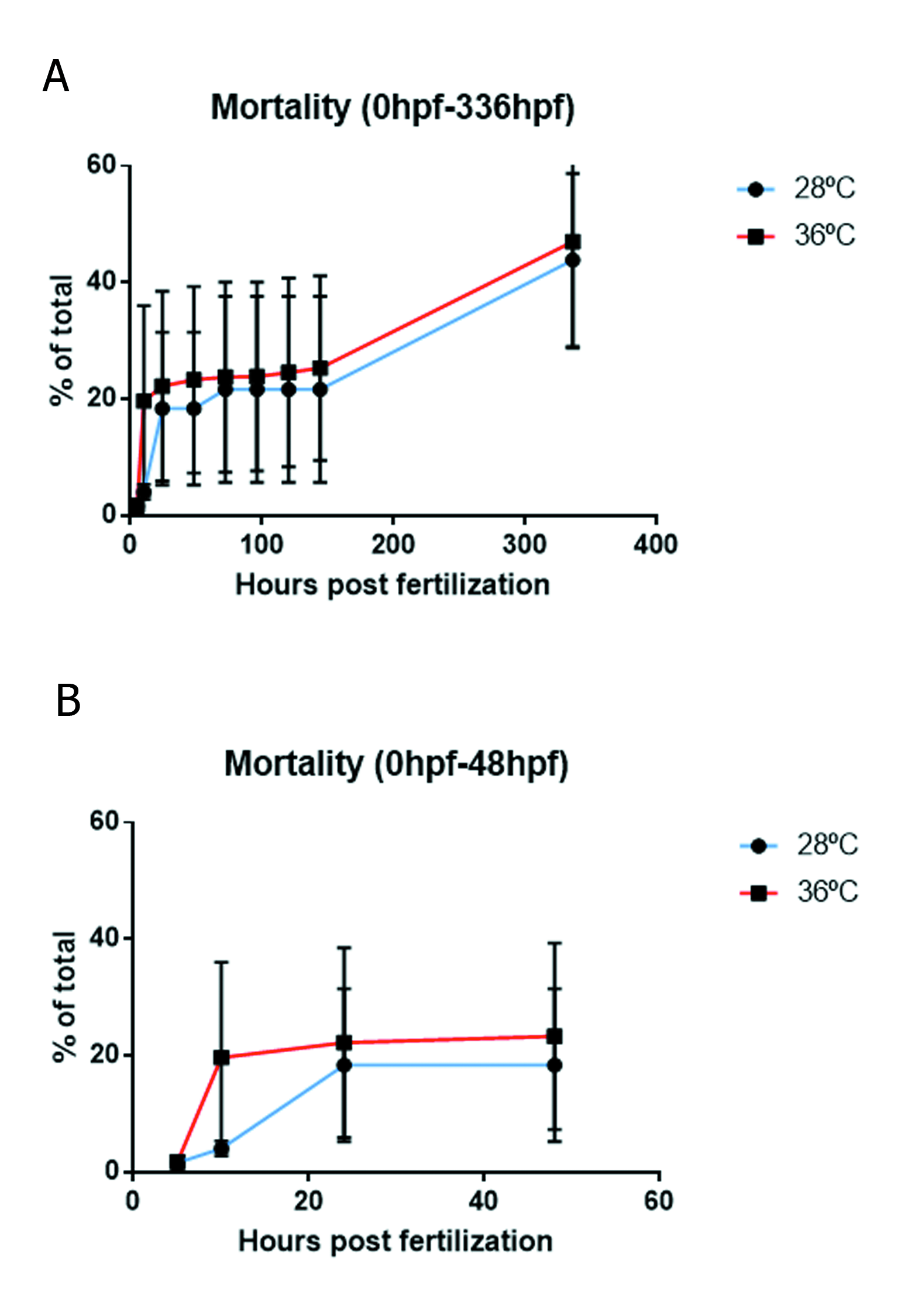

Supplement: Supplementary file 1 [file genes-12-00113-s001.zip › CabezasSainzFigS2.tif]

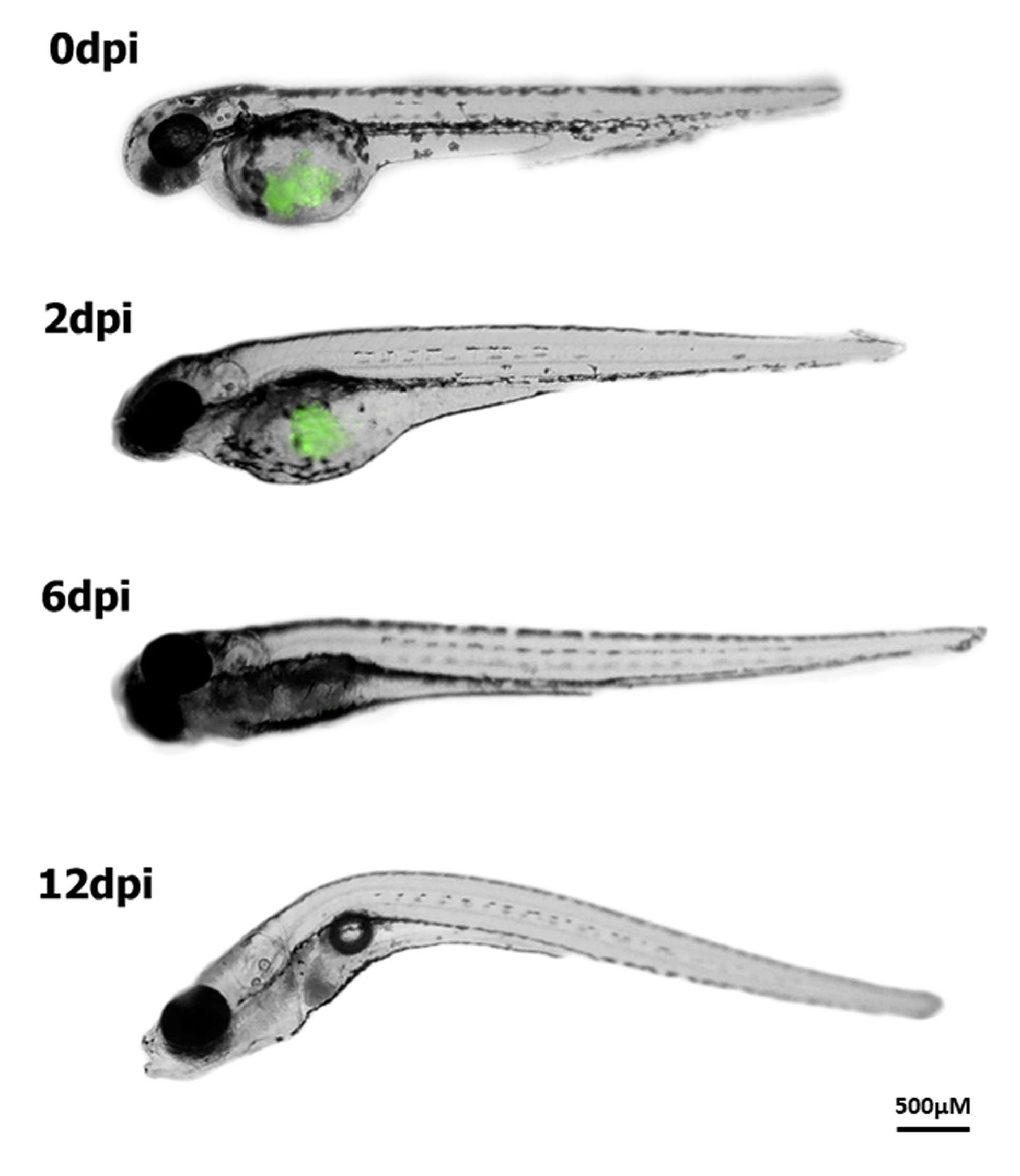

Supplement: Supplementary file 1 [file genes-12-00113-s001.zip › Cabezas-SainzFigS3.tif]

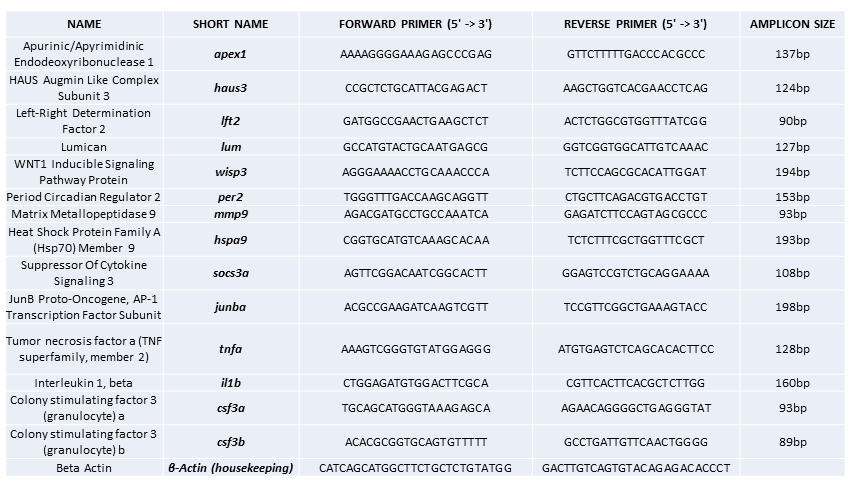

Supplement: Supplementary file 1 [file genes-12-00113-s001.zip › Cabezas-SainzTableS1.tif]
